# Human Gut Hosts a Dynamically Evolving Microbial Ecosystem

**DOI:** 10.1371/journal.pbio.0050199

**Published:** 2007-06-19

**Authors:** Liza Gross

The struggle for survival is challenging enough that diverse forms of life manage the feat only with the help of others. In this regard, humans differ little from termites: just as microbes in the termite gut break down cellulose in its wood-based diet, vast throngs of bacteria in the human intestinal tract break down polysaccharides from plants for us. Scientists have made considerable progress in identifying the constituents of the human gut microbiota, but many questions remain about how the intestinal environment has shaped the evolution of our single-celled companions’ genomes.

In a new study, Jian Xu, Michael Mahowald, Jeffrey Gordon, and their colleagues sequenced the genomes of two bacterial species residing in the human lower intestine to shed light on the forces shaping their evolution. By analyzing the functions and likely origin of genetic features suited to intestinal life, the researchers show that microbes gain the genetic diversity necessary for adapting to their gut habitat in part from other microbes.

Most of the hundreds of bacterial species in the human gut belong to the Bacteroidetes and Firmicutes divisions (which also happen to figure prominently in the termite gut). The researchers focused on two divergent human gut–derived Bacteroidetes species, *Bacteroides vulgatus* and *B. distasonis*. The *B. vulgatus* genome encodes roughly 4,000 predicted proteins, while *B. distasonis* harbors about 200 less. Interestingly, many of these genes were recently duplicated into multigene families, which was surprising because multigene families are relatively rare in bacteria.

To figure out which of these genes might help exploit the nutrients present in the lower intestine, the researchers analyzed five other sequenced *Bacteroidetes* genomes, including two non-gut species: one that lives in soil, the other in the human mouth. Most genes shared by gut and non-gut bacteria play a role in essential metabolic processes and were likely present in their common ancestor. Most genes unique to the gut *Bacteroidetes* function in pathways for polysaccharide metabolism, sensing and responding to environmental cues, and membrane transport. The distribution of these genes, however, suggests that each species has evolved its own strategies for sensing, regulating, and degrading intestinal polysaccharides.

With the help of the well-annotated *B. thetaiotaomicron* genome, Xu et al. identified unique features of the two newly sequenced *Bacteroidetes*. While *B. thetaiotaomicron* can harvest polysaccharides (also called glycans) from multiple sources (plants, host mucous, as well as the outer surfaces of epithelial cells that line the gut), *B. vulgatus* and *B. distasonis* lack the enzymes required for such cosmopolitan tastes. Both species, however, can use glycans derived from their human host, and *B. vulgatus* has a large set of enzymes capable of metabolizing pectins from fruit.

Understanding the forces that shaped the Bacteroidetes genomes provides clues to the mechanisms underlying specific adaptations to their human gut habitat. Toward that end, the researchers constructed phylogenetic trees that reflect the evolutionary relationships of individual kinds of protein-coding genes, to identify genes acquired “laterally,” from other species of bacteria. Their analysis suggested that about 5% of the genes in each genome were acquired in this manner.

**Figure:**
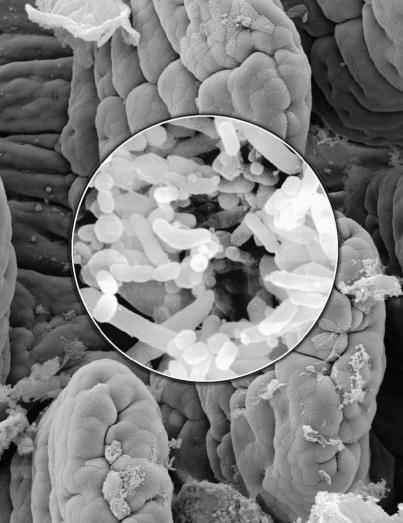
Scanning electron microscope images of *B. thetaiotaomicron*, a prominent human gut bacterium, and the intestine.

A number of these laterally transferred genes in gut Bacteroidetes have been deposited in regions of the genome (*capsular polysaccharide synthesis*, or *CPS*, loci) that encode the machinery involved in manufacturing their surface polysaccharide capsules. Accumulation of diverse *CPS*genes would provide the diversity that these organisms need to adapt to shifting environmental conditions within their gut habitats, including the capacity to adapt to host immune responses directed against their surface carbohydrate structures.

Many of the duplicated (amplified) genes identified  in the newly sequenced gut-dwelling Bacteroides play important roles in sensing, acquiring, and degrading otherwise indigestible polysaccharides in the diet—providing the organism with the capacity to feast on the complex sugars that they encounter in the very dynamic intestinal environment.

What does it take to survive in the gut? For bacteria, survival requires enough genetic diversity, created by forces like lateral gene transfer and gene duplication, to carve out specific nutrient niches and to resist infection by viruses that attack microbes (bacteriophages). The researchers also hypothesize that persistence into the next generation of hosts requires enough functional redundancy in a microbial community that, if a virus were to wipe out a particular species of bacteria, other species could provide the same services to the host.

By showing how the intestinal microbiome—the collective genomes of a microbial community—has responded to its constantly changing environment with dynamic changes in genome structure, this study raises several questions for future research. Humans are exposed to incredibly varied environments—do environmental exposures give rise to individualized “microbiomes?” Might such individual variations overlay a shared core human microbiome? How might personalized microbial communities affect our risk of disease? With efforts under way to sequence the human microbiome, future studies can explore these and many other questions. In doing so, the researchers hope other investigators take a “transcendent view” of human biology that considers the dynamic contributions of our microbial partners to human evolution.

